# The impact of congestion charging on social capital^☆^

**DOI:** 10.1016/j.tra.2017.01.018

**Published:** 2017-03

**Authors:** Luke A. Munford

**Affiliations:** Manchester Centre for Health Economics, University of Manchester, UK

**Keywords:** Social capital, Congestion charging, Policy evaluation

## Abstract

We analyse a new data set to examine how congestion charging policies affect an individual’s investment social capital. We exploit a (quasi-) natural experiment - the implementation of the Western Extension Zone (WEZ) to the London Congestion Charging zone in 2007. We measure investment in social capital by using the frequency of visits to friends and family before and after the implementation of the WEZ. Using longitudinal data collected in January and November 2007 made available by Transport for London, we perform difference-in-difference analysis, using both OLS and interval regression, with the treatment group defined as those who used a car to make visits pre-WEZ. We observe large and statistically significant reductions in visits as a result of the WEZ, with, for example, a reduction of around 20 visits a year to friends. The effect of the WEZ on the number of visits to act as an informal carer is much larger, with reductions of around 100 visits a year. Given that the changes occurred in such a small time frame (10 months), we conclude that the WEZ is likely to be the main driver of these reductions.

## 1 Introduction

A key concept in welfare economics is the principle that individuals should directly pay for the externalities and costs they impose on others. This principle ensures that individuals have an incentive to use the available resources more efficiently. This concept can be easily applied to private motor vehicle use, and in fact urban traffic congestion is a well cited as an example of this principle (Small and Verhoef, 2007). Transport economists have long advocated the use of congestion charging policies to encourage the use of more efficient transport systems, whilst simultaneously addressing congestion and pollution problems. The overall outcome should be the provision of a net benefit to society. However, the vast majority of the existing evidence ignores the fact the these policies could damage both an individual’s level of social capital and their decision to invest in activities which could create social capital, and thus indirectly affect their underlying welfare or utility. We attempt to rectify this omission by examining the impact that the London Congestion Charge (LCC) had on Social Capital (SC).

### 1.1 Background to the London congestion charge and the western extension zone

The LCC was introduced in February 2003 by the then Mayor of London, Ken Livingston, with the aim of reducing both congestion and pollution. Banister (2003) states that:

“*Congestion Charging in Central London is the most radical transport policy to have been proposed in the last 20* *years…*”.

Banister (2003, p. 259).

**Fig. 1:**
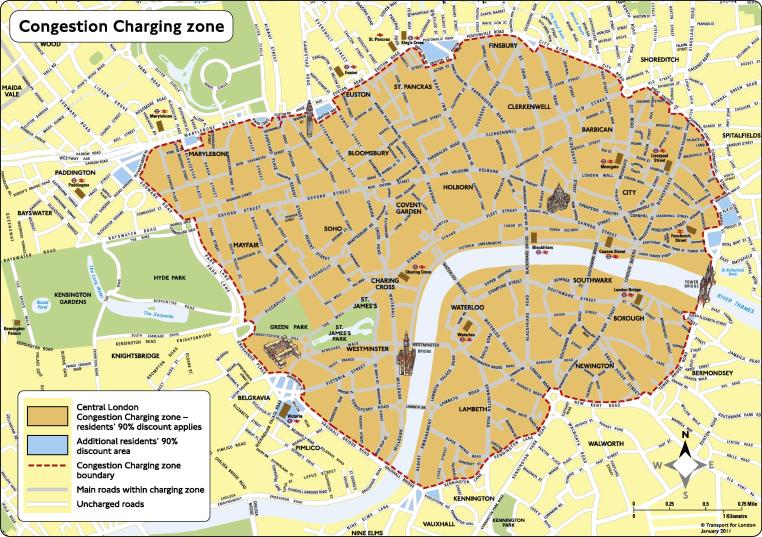
The area covered by the initial (and current) Congestion Charging Scheme.

The area covered by the congestion charge (CC) can be seen in Fig. 1. To drive a vehicle into (or through) the shaded (orange) area in Fig. 1 cost drivers £5 per day in 2003. The price steadily increased to £8 in April 2005, and at the time of writing (2017) stands at £11.50. The fee is applicable between 07:00 and 18:00 Mondays to Fridays, excluding public holidays. The charge is paid for driving, or parking, a vehicle inside the LCC zone, irrespective of the length of time the vehicle is inside the zone.

**Fig. 2:**
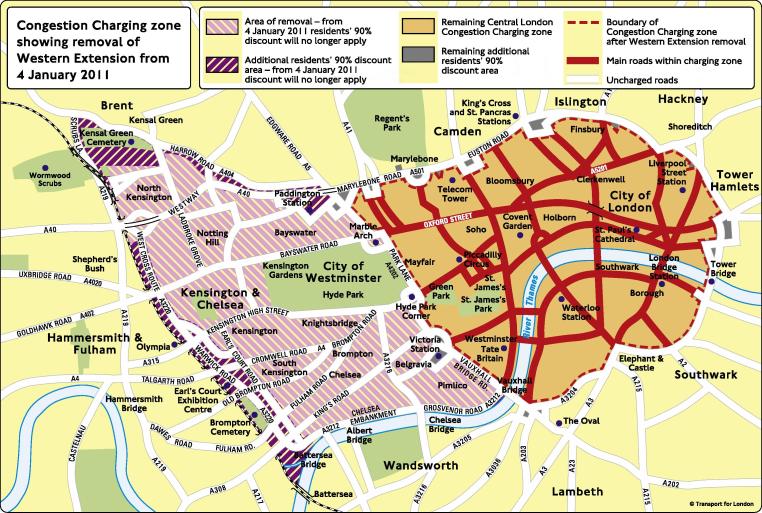
The additional area introduced (and subsequently removed) known as the Western Extension Zone.

Prior to the 2004 mayoral elections, proposals were drawn up to consider expanding the zone to include boroughs to the west, to include the more residential areas. The new, larger, zone would include an additional 80,000 residents, taking the overall number of affected individuals to around 230,000. Some two and a half years after the initial consultation, in February 2007, the Western Extension Zone (WEZ) was formally implemented. Fig. 2 shows the new boroughs included. The extended CC scheme operated as one complete zone; the same charges, discounts and exemptions applied. Following public consultation, the WEZ was subsequently removed in January, 2011 after 62% of respondents backed the removal (Transport for London, 2010).

### 1.2 Aims

The WEZ provides an ideal opportunity to examine the impact that congestion charging has on activities that can lead to the formation or destruction of SC (such as visiting friends and family for social reasons). The aim of this paper is therefore to analyse new data to look for changes in the frequency of these activities which could create/destroy social capital brought about by the introduction of the WEZ. To our knowledge, this paper is unique in two regards: (i) this is the first empirical piece of work to analyse this particular data set in an academic context; and (ii) this is the first analysis to examine the *ex post* impact that congestion charging has on social capital.

The main mechanism that explains why we expect the LCC to lead to a reduction in social capital is the associated costs. Not only are there monetary costs to drive a vehicle through the affected area, but there are also time and administration costs (such as paying the fee). However, if individuals can substitute between mode (*i.e.* switch from car to public transport) then there may not be a significant reduction is social capital.

The paper proceeds as follows: Section 2 will provide a literature review; Section 3 outlines the available data and methodology; Section 4 presents the results obtained and provides a discussion of these; and Section 5 concludes.

## 2 Literature review

The aim of this paper is to investigate what impact road pricing policies have on social capital (or social capital forming/reducing activities), something which we believe has not been done before. Therefore this section is split into two component sub-sections: the first will briefly review the growing body of empirical literature that examines social capital; and the second will look at previous studies that evaluate existing congestion charging schemes. A small subsection of the existing literature is concerned with the *ex-ante* evaluation of road pricing, with regards to social inclusion/exclusion - a not wholly dissimilar concept to social capital. However, our area of focus in this analysis is an *ex-post* evaluation.

### 2.1 Social capital

Social capital (SC) is a term whose origins are in sociology and psychology. No precise definition exists, but in his synthesis Woolcock (1998) defines social capital as:

*“… a broad term encompassing the norms and networks facilitating collective action for mutual benefit.”*

Woolcock (1998, p. 55).

Portes (2000) further examines the origins of social capital and looks at its applications in modern sociology and psychology. He argues that social capital encompasses all that is good about sociability and hence has a place in sociological theory, but concludes, however, that *“excessive extensions of the concept may jeopardize its heuristic value.”* (Portes, 2000 pp. 43). In a sociological setting, Coleman (1994) points out that social capital is an ‘*intangible concept*’, which is a view held by many sociologists. Because of this perceived intangibility, the majority of the sociological work on social capital has focused on the conceptual understanding, as opposed to actually measuring it and determining how individuals can influence their stock of social capital.

One of the most cited works on SC in recent years is Putnam (2000) - the influential *Bowling Alone: The Collapse and Revival of American Community*. In this book, Putnam argues that, amongst other things, changes in commuting/transport behaviour have had a detrimental affect on how Americans engage with each other, *i.e.* reduced their stocks of SC. Putnam (2000) argues that there are two distinct strands of social capital: (1) bonding -or exclusive- SC; and (2) bridging -or inclusive- SC. The former relates to strong social ties between homogenous individuals (*i.e.* within families and/or existing networks of friends) whereas the latter is concerned with attempting to expand social networks to include a more diverse social grouping. As a result of these two separate strands of SC, Putnam uses many instruments for SC, including, most notably the uptake of bowling in social and competitive environments. He also uses data on the frequency of visits to friends and family, which to some extent validates our choice of SC proxy.

Islam et al. (2006) systematically reviewed the literature on social capital, building on the two definitions Putnam proposed. They further include structural and cognitive components to social capital, where structural refers to the density of social networks and cognitive relates to an individual’s perception of (amongst other things) levels of interpersonal trust and reciprocity.

Steven Durlauf has written comprehensively on the use of social capital as a socioeconomic indicator for an individual. Durlauf (2002) argues that the exact definition of social capital is ambiguous in the majority of empirical studies, and as such no true causal relationship can actually be identified. He further argues that this has led to a variety of disparate ideas emerging within the social capital literature. Durlauf and Fafchamps (2004) extend further this argument by accusing social capital of possessing ‘*conceptual vagueness*’. However, despite its limitations, Durlauf and Fafchamps (2004) do conclude that social capital is an important concept in social science research, and its determinants should still be investigated.

Kan (2007) propose further extensions to social capital by including spatial dimensions. He proposes social capital be broken down into local social capital (local SC) and distant social capital (distant SC). Local SC includes friendship ties with individuals living nearby, and may be beneficial to the wider local community by (amongst other things) reducing crime rates and improving the local physical geography of a neighbourhood. Alternatively, distant SC can be thought of as family and friends living far away from an individual, and hence reducing the possible benefits of strong social ties. Kan exploits these differing spatial aspects of social capital to look at residential mobility, conjecturing that high levels of local social capital are likely to reduce the geographic mobility of an individual. Using the Panel Survey of Income Dynamics, especially questions like those mentioned in the paragraph above, he finds that (as expected) high levels of local social capital deter people from moving, hence reducing residential mobility.

In addition to the above, the factors which determine an individual’s level, or stock, of social capital has been the focus of much recent empirical work in the economic literature. However, as no precise definition exists, many proxies have been used. For example Alesina and La Ferrara (2000) use participation in voluntary groups in the United States as a proxy and find that the racial make up of a groups plays a great role in determining who participates in that group. Kan (2007) analyses American data and proxies social capital with people’s beliefs on how friendly their neighbourhood is.^1^ They deduce that higher levels of local social capital imply residents are less likely to be residentially mobile.

For more detail discussion on social capital see, amongst others, Durlauf and Fafchamps, 2004, Islam et al., 2006, Putnam, 2000.

#### 2.1.1 Social capital and social inclusion/exclusion

The concepts of social capital and social inclusion (or exclusion) can often become very hard to disentangle. The terms are often used interchangeably due to the difficulties in pinning down each term to a strict definition (Daly and Silver, 2008). In this paper, we think of the social inclusion being a key driver of social capital, with both being determined by social networks and how connected a person is Cattell (2001). We proxy social capital here by the number of visits made to friends and family. This definition (or proxy) has been considered before in the literature by, *inter alios*, Putnam, 2000, Portes, 2000, Cattell, 2001.

No empirical papers have, to our knowledge, studied the impact that congestion charging policies have on social capital. Hence this study is unique as it is the first to do so. Some related research topics have been briefly touched upon in the empirical literature, and these are summarised in the next subsection.

### 2.2 Congestion charging policies

The LCC has been examined in relation to a number of key economic areas. For example, Schmöcker et al., 2006, Quddus et al., 2007 both find that the CC reduces revenues at a large department store; and Zhang and Shing (2006) find that the gap in house prices between houses just inside and just outside the LCC zone actually falls (they hypothesise a widening gap).

There is consistent evidence that congestion charging schemes reduce pollution and hence reduce mortality. For example, Tonne et al. (2008) show that the London CC reduced both nitrogen dioxide (NO_2_) and particulate matter, up to size ten (PM_10_) in wards affected by the CC zone when compared to non-affected areas of London. The authors estimate that these reductions in pollution will lead to 183 life years gained per 100,000 population for those who live within the affected zone, compared to 18 years per 100,000 for non-affected Londoners. Gibson and Carnovale (2015) consider a ‘natural experiment’ brought about by an unanticipated court injunction in Milan (where road pricing was temporarily suspended for 8 weeks), and they too show that road pricing does lead to a reduction in air pollution.

Green et al. (2016) consider the effects of the London CC on both the number and severity of traffic accidents. Using a rich dataset of all motor vehicle accidents reported to the police in Britain between 2000 and 2009, and applying difference in difference techniques (both with real and synthetic control groups), they find that the LCC did lead to a reduction in accidents, both in the CC cordon, but also in adjacent areas of London too. The results were robust to considering times of the day where the charge was not applicable, and also considering vehicles that were exempt from the charge.

In addition to the literature relating to the LCC, several further studies have looked at the introduction of the Stockholm Congestion Charge (SCC) scheme. Eliasson et al. (2009) provide an overview of the effect of the SCC, noting that the SCC had reduced congestion far and above the expected levels. They further argue that the SCC resulted in *“favourable economic and environmental effects”* (Eliasson et al., 2009 p240), including positive effects on both the regional economy and on retail. Karlström and Franklin (2009) look at mode choice due to the SCC, and find people are likely to switch away from cars. They further look at inequalities by examining distributional effects, and find that it is most poor and the most rich who are more likely to switch. Finally, Schuitema et al. (2010) compare and contrast the opinions of residents of Stockholm before and after the implementation of the SCC. They find that residents are actually more in favour of the SCC *ex-post* than they thought they would be *ex-ante*.

#### 2.2.1 The effects of congestion charging on social exclusion

Whilst the impact that congestion charging has on SC, *per se*, has not been examined in the literature, its impact (or *expected impact*) on related concepts has been. For example Bonsall and Kelly (2005) argue that if congestion charging was introduced in the UK city of Leeds, the impact would depend on the precise definition of the charge area, as well as on the charges and exemptions provided. They examine six hypothetical policies in turn. Their main area of focus is what they call ‘at risk groups’. These people are already among the most socially excluded within the city, and include low income individuals, and disabled individuals. They argue that any CC policy would place these people into higher levels of social exclusion, especially those on low incomes with no realistic public transport alternatives to the car journeys they make. They conclude that the policy with the less serious consequences for social exclusion is a policy based on charges proportional to the distance driven within any charge area.

Similar to the above, Rajé (2003) examines what could happen to social exclusion in Bristol (UK) following a CC policy. She argues that it may be possible to promote social inclusion, and hence reduce social exclusion, if the monies raised from a congestion charge are used to improve current conditions - including enhancing public transport - and to promote public transport usage.

Whilst the studies of Bonsall and Kelly, 2005, Rajé, 2003 provide useful insights as to what may happen as a result of a congestion charge policy, they are both *ex-ante*. The evaluation we propose here is *ex-post* as it is based on an evaluation of a CC policy that had already been implemented.

## 3 Data and methodology

### 3.1 Data

This paper will analyse unique data provided by Transport for London (TfL). A panel survey consisting of five waves was commissioned to canvas public opinion on the WEZ. A random sample of representative individuals was contacted by an initial telephone call in which they were asked if they would like to participate in further waves of the main survey. If they agreed they were then contacted, if possible, in all five waves. The telephone interviews relating to specific waves were carried out approximately 3–4 months apart. Table 1 shows the approximate dates of the interviews and the number of respondents, along with the response rate as a percentage of the initial sample.

The total number of individuals in the analysis that will follow is 1312 - *i.e.* all those people in wave 2 who are remaining in wave 4. However, not all questions are applicable to all individuals, hence sample size varies by outcome. As we utilise difference in difference methodologies, we require observations both before (wave 2) and after (wave 4) the policy was implemented. The fact that waves 2 and 4 are only c. 10 months apart means that any results found here are likely to be driven by the implementation of the WEZ. Such a relatively small time frame would indicate that other confounding factors may not have had sufficient time to ‘kick-in’ (an Internet search showed that there were no major travel problems in London during the time frame considered here. There were storms and snow in January 2007, although these were not overly severe, and they were prior to the implementation of the WEZ).

Table 1 provides a general description of the type of questions asked in each wave. Waves 2 and 4 both have detailed information on the frequency of visits to friends and family. We argue that these variables are good measures of activities which can lead to the formation of new social capital (as well as increase existing levels). The data in wave 2 are concerned with before the WEZ was implemented, whereas wave 4 relates to during the tenure of the WEZ.

In waves 2 and 4 everyone in the survey is asked: *“…in the western extension zone, between 7* *am and 6* *pm on weekdays, how often do you…?”* and were given five different questions:

(i) *“Visit family members who live in the western extension zone in their home”*;
(ii) *“Meet up with family members who live in the western extension zone at a location in the WEZ other than their home”*;
(iii) *“Visit friends who live in the WEZ in their home”*;
(iv) *“Meet up with friends who live in the western extension zone at a location in the WEZ other than their home”*; and
(v) *“Visit someone at their home in the WEZ as a carer/volunteer ”*.

After the above questions, respondents are also asked to indicate *“how do you normally travel to make that journey?”*, with possible responses: (a) car, (b) public transport, (c) taxi, (d) walk, or (e) cycle.

We classify (i) and (ii) as visits to family, and (iii) and (iv) as visits to friends. The question about caring made it clear these should be visits over and above general social visits.We also know whether or not the respondent is a WEZ resident, and hence we can break down visits to WEZ residents into two groups: (1) those made by fellow residents and (2) those made by non-residents. We would not expect homogeneous effects across these two groups as we believe visits by fellow residents may be more resilient to change due to the local SC hypothesis proposed by Kan (2007). We also thank an anonymous reviewer for pointing out that WEZ residents were entitled to apply for a discount of up to 90% off the cost of driving a vehicle through the zone.

Then additionally those individuals who lived within the WEZ were asked the same questions, only relating to the visits that they make to friends and family who live outside of the WEZ. This gives us information on three types of visit: (1) WEZ residents to WEZ residents; (2) non-residents to residents; and (3) residents to non-residents. We additionally pool groups (1) and (2) so we can consider all visits made to residents.

The responses to all ten questions above were coded on a ten point ordinal scale, where (1) corresponded to a response of 5 days a week or more, through to (9) which indicated never. Each ordinal value represented an interval of visits (Table 2). (10) was not applicable. Hence, lower scores indicate a higher frequency of visiting friends and relatives. We recode the responses so higher values relate to higher frequencies of visits, and hence higher levels of SC. The new scale is detailed in Table 2. The results based upon these categories, and mid-points, are robust to slight changes in the upper and lower limits.

#### 3.1.1 Other variables of interest

As well as dummy variables for time-period, mode choice, and their interaction (see next section) other socio economic information are included in the regression model. Due to the limited nature of the data, the set of socio-demographic variables is not as rich as would be preferred. However, we include employment status (employee/employer/self-employed/student - the omitted category is not-employed/retired), banded age groups (18–24, 25–44, 45–59, 60–64 - the omitted category is age over 65), gender and an indication if an individual’s income had increased or decreased since wave 2.

### 3.2 Methodology

As a starting point, we compare the average frequency of visits to friends and family before and after the implementation of the WEZ, for both WEZ residents and non-residents. To do this, we use the midpoint of the ranges.

#### 3.2.1 Difference-in-difference

The available data lends itself to utilising difference-in-difference (D-i-D) techniques. Define the outcome of person *i* who uses mode choice *m* to make visits at time *t* as $Y_{\mathrm{imt}}$. The policy intervention here affects the people who use a car to visit their friends and family, and hence individuals who state they use a car to make these visits in wave 2 are defined as the *treated*.

The most efficient way to estimate D-i-D is by applying regression analysis, requiring estimation of the model:

(1) $$Y_{\mathrm{imt}}=\alpha +\beta ({\text{Car}}_{m})+\gamma ({\text{Post}}_{t})+\delta ({\text{Car}}_{m}\times {\text{Post}}_{t})+\psi x_{\mathrm{imt}}+\varepsilon_{\mathrm{imt}}$$

where Car_*m*=1_ if person *i* used a car to visit friends and family in wave 2, and 0 otherwise. Post_*t*=1_ if the data relates to Wave 4 (*i.e.* after the implementation of the WEZ), and 0 otherwise. The vector $x_{\mathrm{imt}}$ in Eq. (1) contains information on employment status, age, gender and changes in income of individual *i* in time *t*.

Interpretation of the parameters in Eq. (1) is as follows: α shows the value of the dependent variable for those who not use a car to make visits before the CC was implemented; α+γ relates to people who don’t drive a car to make visits after its introduction; α+β defines the value of $Y_{\mathrm{imt}}$ for individuals who did drive a car to make social visits before its introduction; and α+β+γ+δ is the value of $Y_{\mathrm{ist}}$ for people who drove a car to visit friends and family after the WEZ was introduced. The vector ψ contains the coefficients for the socio-economic variables.

The coefficient of interest from Eq. (1) is therefore the parameter δ - defined as *the difference-in-difference* parameter; alternatively denoted the *treatment effect*. The main benefit of using regression-based D-i-D is it allows for standard errors to be created and examined, and hence we can test for the statistical significance of the parameters of interest.

#### 3.2.2 Interval regression

Given that the response to the questions relating to frequencies of visits are answered on an ordinal scale, it is methodologically preferable to utilise techniques that recognise this ordinal nature. One such technique is that of Interval Regression (IR), first developed by Stewart (1983). IR is concerned with estimating model parameters when the response categories are a subsection of the real number line (that is there are upper bounds, $u_{k}$, and lower bounds, $l_{k}$, such that $y_{i}=k$ if $l_{k}⩽y_{i}^{*}⩽u_{k}$). In essence, IR is an extension to the ordered probit (OP) model. Recall the OP model is defined such that:

(2) $$y_{i}=k\;\text{if}\;\mu_{k-1}<y_{i}^{*}⩽\mu_{k}\;k=1\text{,}\ldots \text{,}K$$

where here the latent variable, $y^{*}$, is expressed as:

(3) $$y_{\mathrm{imt}}^{*}=\alpha +\beta ({\text{Car}}_{m})+\gamma ({\text{Post}}_{t})+\delta ({\text{Car}}_{m}\times {\text{Post}}_{t})+\psi x_{\mathrm{imt}}+\varepsilon_{\mathrm{imt}}$$

The IR model then fixes the μ terms to take the values reported in Table 2. For example, $y_{\mathrm{ist}}^{*}=9$ implies that respondents make visits 5 days a week or more, which corresponds to between 260 and 365 days a year. These values of 260 and 365 can be imputed as values for $\mu_{7}$ and $\mu_{8}$, such that $y_{\mathrm{ist}}=9$ if $260<y_{\mathrm{ist}}^{*}⩽365$. Because the value of the μ’s are known, the estimates of the τ coefficients are more efficient. It is also possible to identify the variance of the error term, denoted $\sigma^{2}$, and therefore the scale of $y_{\mathrm{ist}}^{*}$ (Jones, 2000).

The likelihood function of the IR model subsumes that of the tobit model. In the case here, all data observations are in an interval or left censored (by zero). Denote observations that are in an interval as j∈I, where observation $y_{j}$ is in the interval given by $\left[y_{1j}\text{,}y_{2y}\right]$. The bounds under consideration here are outlined in the data section. Further denote the left (or zero-) censored observations as j∈L. Then the log-likelihood function is:

(4) $$\mathrm{LL}=\underset{j\in L}{\sum }\log\Phi \left(\frac{y_{L_{j}}-\tau z}{\sigma }\right)+\underset{j\in I}{\sum }\log\left\{\Phi \left(\frac{y_{2j}-\tau z}{\sigma }\right)-\Phi \left(\frac{y_{1j}-\tau z}{\sigma }\right)\right\}$$

#### 3.2.3 Difference-in-difference: Assumptions and possible violations

For the results presented in the next section to be unbiased, D-i-D analysis requires that certain assumptions are met. Firstly it is necessary to assume that the treatment is exogenous, secondly the D-i-D approach assumes a common trend across control and treatment groups, and finally it is necessary to assume that the composition of control and treatment groups is stable over time.

The treatment (the WEZ) should be exogenous in that it only affected those who make visits by car in wave 2. All other modes of transport are unaffected by the introduction of the WEZ, as public transport fees did not change. Similarly, there was no monetary cost to walk or cycle through the WEZ. The common trends assumption is not possible to test here, as data is only available for one period before the intervention (wave 2), and one period after the intervention (wave 4). In this analysis, we define treated on the basis of mode choice in wave 2. However, it is possible that people switch mode between wave (possibly as a direct result of the policy of interest). However we repeat our analysis on a subsample of people who use a car to make visits in both wave 2 and wave 4, and the results are essentially the same as those reported in the results section. Hence we can assume that this possible violation of Assumption 3 is not a major issue.

In Eq. (1) we do not control for macroeconomic variables. Given that the policy affected whole areas of London we assume that the macro effects are constant for the whole of the treatment group and similarly are constant for the whole of the control group. This ensures that the Stable Unit Treatment Value Assumption (SUTVA) (e.g. Cox, 1958, Hudgens and Halloran, 2008) is satisfied here, as the treatment for all of the treated individuals is constant. We further assume that the treatment status of any given individual does not affect the potential outcome for another individual. That is, we assume that the visiting friends and family behaviour of an untreated individual (walkers, cyclists, or public transport users) is unaffected by the visiting behaviour of an individual who travels by car. This assumption may be violated if, say, an individual who walks to visits family has to make more trips to make up for the reduction in trips by a car user. However, as we cannot identify friendships and family ties within our data we cannot test for this, and hence we assume that the assumption is valid. The variable Post_*t*_ will further pick up any unobserved macroeconomic effects in this model set-up.

## 4 Results and discussion

### 4.1 Simple differences

We present the basic differences in the number of visits to friends and family (where we impute the midpoint of the range (Table 2) as the dependent variable) made by any mode of transport in Table 3. The main column of interest is column (4) which shows the changes between waves 2 and 4 $\left(\Delta \overset{¯}{y}={\overset{¯}{y}}_{4}-{\overset{¯}{y}}_{2}\right)$. A negative number in column (4) indicates a reduction in visits. We observe that all type of visits fell between waves, and that 8 out of 10 of these reductions were statistically significant. The biggest raw fall in visits is to friends away from their home, and this is consistent for both visits to WEZ residents, and visits from WEZ residents to non-residents.

We then go on to examine the above differences by treatment status (recall anyone who indicated that they used a car to visit friends or family in wave 2 are the ‘treated’, and every other mode of travel is the control group). We present these differences in Table 4, and also pictorially in Fig. 3a, Fig. 3b. From both the Figures and the Table, we observe reductions for both control and treatment groups for all visits, and that it is the treatment group that have the greatest reduction. Examining Table 4 shows that 8 out of 10 of the difference-in-difference coefficients (column (7)) are statistically significant, and that it is visits to act as a carer that suffer the most. As an example of interpretation, the last row indicates that the WEZ lead to a reduction in the number of visits made by a WEZ resident to act as a carer to fall by 103 times a year. This is a huge difference, and is statistically significant (p<0.001).

### 4.2 OLS difference-in-difference

The results presented in Table 4 do not control for additional socio-economic information, and we turn to these results now. We start by presenting the OLS D-i-D results.

The results for visits to family, presented in Table 5, show that after the introduction of the WEZ visits fell. However, this reduction is not usually significant. We also observe that individuals who used a car to make visits were much more likely to make more visits. For example, in column (1) we observe that car drivers make, on average, 45 more visits a year than non-car drivers. The main row of interest is the interaction row (After × Car), from which we obtain the difference-in-difference coefficient.

The WEZ did not have an impact on the pooled number of visits to family members who lived within the WEZ cordon (column (1)). However, when we split these visits down by who made them (WEZ residents or non-residents), we observe that the number of visits made by non-residents (column (3)) fell by 37 visits a year (*p* < 0.01), but there was no statistically significant reduction in visits made to fellow residents. When we consider visits made be residents to non-residents, we observe a significant reduction of 12 visits per year (p<0.05).

When we consider all visits to family members who live within the WEZ at locations away from their home (column (5)), there is a statistically significant reduction of 17 fewer visits per year (p<0.05). We also observe a reduction in the number of visits made by residents to family members who live outside of the cordon (column (8)).

In Table 6 we consider visits made to friends. Here we see fewer visits being made after the implementation of the WEZ (particularly at locations away from their home), and again that car drivers make statistically more visits than non-car drivers. When we consider visits made to the home of friends, we observe statistically significant reductions in visits to friends who live inside the cordon, both from fellow residents (reduction of 17 visits a year, p<0.05; column (2)) and from non-residents (reduction of 18 visits a year, p<0.05; column (3)). There is no statistical difference in visits made by residents to non-residents (column (4)). When we look at visits made to friends away from there home, we observe statistically significant reductions in all types of visits.

We then consider visits made to act as an (informal) carer (Table 7). Here the effect of the WEZ is massive. For example, in column (1) we observe that the WEZ caused 79 fewer carer visits a year (p<0.05) to individuals who lived within the WEZ. When we break this down by who is making the visit, we can see that non-residents make fewer visits, but the effect is less statistically significant when compared to visits by fellow residents. Column (4) shows that the WEZ caused a reduction of 104 visits a year by WEZ-residents to provide informal care to non-WEZ-residents (*p* < 0.01). These numbers are very large, and inspection of Fig. 3a, Fig. 3b would indicate these massive reductions are being driven by the large reductions in the number of visits being made by the treatment groups (*i.e.* by car drivers).

What is interesting to note from all three of the above Tables is that (changes in) income is rarely a statistically significant determinant of the number of visits made. This would suggest that it may not be the financial costs of the WEZ that are causing the reductions in visits. If the main reason was about the cost, we would expect to see negative and significant coefficients on the ‘reduction in income’ variable. However, given the imprecise point estimates, coupled with the grouped nature of this variable, this explanation is somewhat speculative. We cannot assert if income had increased by £10 or doubled say In general, younger people make more visits, as do the employed, and the effect of gender is ambiguous.

It is also worth noting here that when we break down the number of visits to people who live within the WEZ cordon by who makes them (fellow residents or non-residents), we consistently observe larger reductions in the number of visits made by non-residents. We thank an anonymous reviewer for highlighting this, and providing a possible explanation. Individuals who lived within the zone were allowed to register for a discount of up to 90%. This means that for them the financial costs of making visits by car may not be as large. However, if this financial difference was the only reason for this, we would expect to observe no statistically significant reduction for visits by residents to other resident, or visits by residents to non-residents. This is not the case, as we do observe significant, and often quite large, reductions in both of these types of visit. We propose another explanation is that local SC is more resilient than distant social capital (Kan, 2007).

### 4.3 Interval regression difference-in-difference

As mentioned above, it is theoretically preferable to model the responses using Interval Regression, due to the underlying ordinal nature of the outcome variables.

For visits to family (Table 8) we observe essentially the same results as in the OLS case. That is after the implementation, there were fewer visits and that car users make more visits compared to other modes. The difference-in-difference terms are again typically bigger in magnitude and more significant for visits away from home. For example, the WEZ caused a reduction of 15.5 visits a year to family to people who live inside the WEZ (*p* < 0.01, column (5)). As with the OLS case, we observe that visits made by non-residents to residents reduced much more than visits by residents to other residents. The difference-in-difference coefficients are smaller in the interval regression case than they are when we modelled using basic OLS.

Table 9 considers visits to friends using interval regression. Similar to above, we observe very similar results as to when we utilised OLS. However, in the IR case the difference-in-difference coefficients are all negative and significant, whereas in the OLS case not all coefficients were significant.

Finally, we consider the impact that the WEZ had on caring in an IR model (Table 10). Similar to the OLS case we observe really large and significant D-i-D terms, and the IR coefficients are slightly larger than the OLS ones. For example, the WEZ lead to a reduction in 118 caring visits a year (*p* < 0.01, column (4)).

### 4.4 Changes in mode of travel to visits

One possible explanation for the results presented in this study (discussed in Sections 4.2, 4.3) could be that individuals who used a car to make the journey to visit friends and family in wave 2 (the ‘treatment group’) may simply switch to an alternate mode of travel in wave 4. To examine this possibility, we present the transition matrix, conditional on being a car driver in wave 2 (Table 11). We observe that for all ten of the types of visit considered, car remains the modal choice in wave 4. However, some categories (such as visits to both friends and family away from their home) experience bigger reductions in car drivers when compared to, say, visits as a carer. These results would suggest there is some mode resilience, but it is stronger from certain categories.

### 4.5 Limitations

This work may be limited by the available data. The first potential limitation is the definition of social capital used here. We proxy social capital using the frequency of visits made to friends and family. This is likely to be only a small part of an individual’s true level of social capital. In fact it is likely to be a proxy for activities which create social capital, not social capital itself. However, this is the only variable we have available. The fact that these variables have been used before as a proxy measure before (e.g. Putnam, 2000) somewhat alleviates this issue.

The second limitation is the fact that information on the frequency of visits made by non-residents to other non-residents is not recorded. These types of visits are not subject to the congestion charge, and hence we could have used them (people who use cars to make non-resident to non-resident trips) as a true control group in a difference-in-difference-in-difference methodology.

## 5 Conclusions

This paper provides a unique insight into the *ex-post* effect that congestion charging policies have on social capital. Difference in difference techniques, based on a number of model specifications, are employed to evaluate the impact that the Western Extension Zone of London’s Congestion Charging scheme had on the frequency of visits to friends and family.

When analysing simple differences, it is noticeable that after the implementation of WEZ the frequency of visits fell. The fact that the time period under consideration (c. 10 months) is relatively small adds weight to these results. In a longer time frame, it is possible that other confounding factors may have contributed to this marked drop in visits. However, such a small time frame would seem to imply that the WEZ in the main driver of these results.

When looking at raw D-i-D techniques, we observe that those who used a car to make visits prior to the implementation of the WEZ make substantially fewer visits after it is introduced, when compared to people who use other forms of travel. These results are robust to controlling for available socio-economic information, including age, gender, occupational status, and changes in income. We further observe that those who initially drove a car make, on average, more trips than other individuals.

The choice of methodology (OLS vs. Interval Regression) does not seem to greatly affect the results. In the majority of cases both methods give similar effects in terms of magnitude and significance. However, we argue here that as IR is theoretically more justified, and easy to implement, then this should be the method of choice.

When considering visits at a person’s home versus away from their home, we consistently observe bigger reductions for visits away from home. However, the WEZ also led to significant reduction to visits to people at their homes, only not as large in magnitude.

The implementation of the WEZ had the greatest impact on the frequency of visiting friends (in terms of magnitude and significance), when compared to visits to family. However, as above, visits to family were also statistically significantly reduced as a result of the WEZ.

What is most startling about the results presented here is magnitude of the reduction in visits to act as a carer. This is consistent for both visits to people within the WEZ and for visits by WEZ residents to care for non-residents. In all cases, we observe statistically significant reductions of around 100 visits a year. This large reduction may lead to a higher demand for more formal healthcare services, such as doctors and hospitals.

From the analysis presented here, it would appear that the Western Extension Zone did reduce the number of times an individual visited their friends and/or family. At a time when the importance of social capital and social inclusion is becoming more prominent, these are interesting results. Further analysis, with more detailed data, is required to understand the mechanisms behind these findings.

## Figures and Tables

**Fig. 3a:**
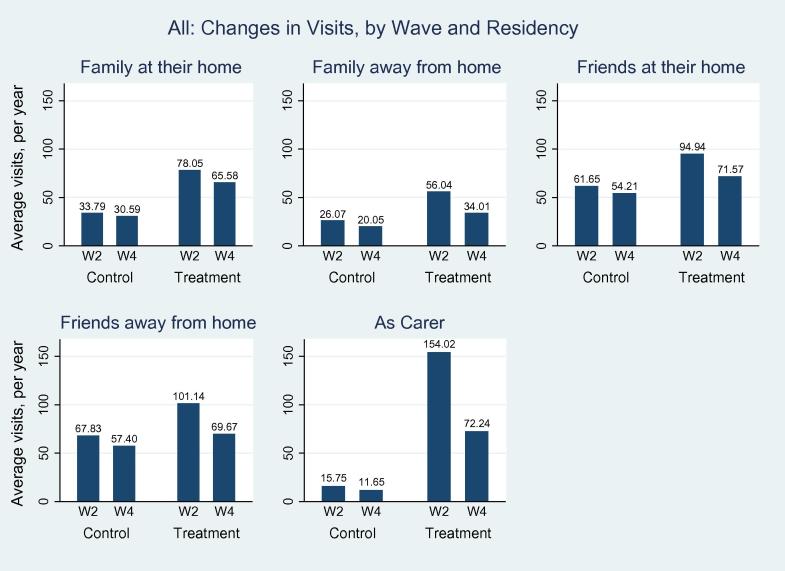
The effect of the WEZ on visits to people who live within the charging zone.

**Fig. 3b:**
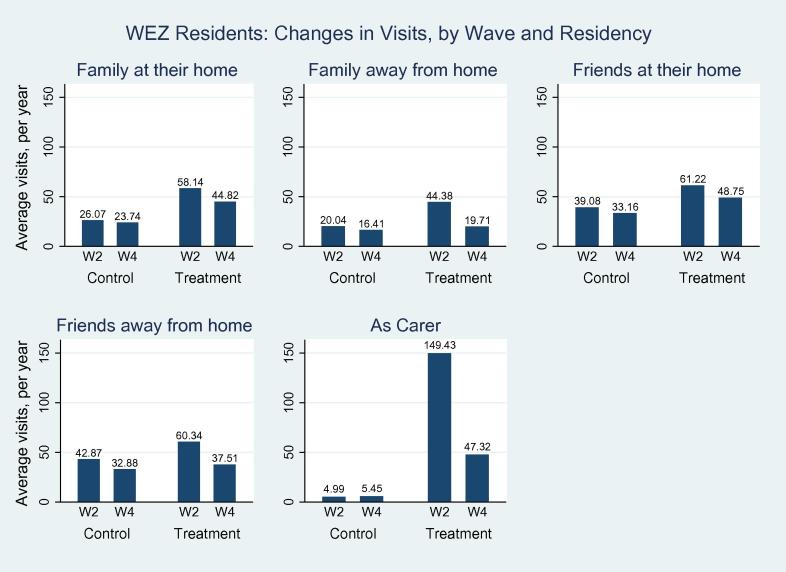
The effect of the WEZ on visits to people who live outside of the charging zone from people who live within the zone.

**Table 1:** Details of the five waves of available data.

Wave	Date	Respondents (%)	Questions asked
1	c. 09/2006	4021 (100%)	Socioeconomic information and expectations
2	c. 01/2007	2437 (61%)	Frequency of visiting friends and family (F&F)
3	c. 05/2007	1755 (44%)	Shopping and childcare information
4	c. 11/2007	1312 (33%)	Frequency of visiting F&F
5	c. 02/2008	939 (23%)	Incomplete

*Estimation data set sample size:*N=1,312			

**Table 2:** The possible responses, and recoding, of the dependent variables.

Raw value	Response	Mid-point^a^	Range of possible values^b^
1	Never	y=0	y∈[0,0]
2	Less than once or twice a year	y=1	y∈(0,2]
3	Once or twice a year	y=4	y∈(2,6]
4	Every few months	y=9	y∈(6,12]
5	Every month or so	y=18	y∈(12,24]
6	A few times a month or so	y=38	y∈(24,52]
7	1–2 days a week	y=104	y∈(52,156]
8	3–4 days a week	y=208	y∈(156,260]
9	5 days a week or more	y=312.5	y∈(260,365]

a These values are used in the midpoint OLS models.

b Traditional notation is employed, such that x∈(a,b]⇒a<x≤b. These values are used in the Interval Regression models.

**Table 3:** Average number of visits: pooled and by wave.

	Means			Diff.	Diff.
Outcome	Pooled	W2	W4	W4-W2	p-value
Visits to Family at their home a	39.38	42.12	36.68	−5.44	p=0.1125
Visits to family away from their home a	27.04	31.63	22.45	−9.18^∗∗∗^	p=0.0003
Visits to family at their home b	32.27	35.26	29.36	−5.90^∗^	p=0.0601
Visits to family away from their home b	22.05	26.98	17.29	−9.69^∗∗∗^	p=0.0001
Visits to friends at their home^a^	63.39	68.87	57.92	−10.95^∗∗∗^	p=0.0006
Visits to friends away from their home a	67.53	75.00	60.02	−14.98^∗∗∗^	p<0.0001
Visits to Friends at their home b	41.68	45.59	37.71	−7.88^∗∗^	p=0.0116
Visits to friends away from their home b	41.16	48.01	34.22	−13.79^∗∗∗^	p<0.0001
Visits as carer a	16.31	19.58	12.92	−6.66^∗∗^	p=0.0142
Visits as carer b	8.23	9.73	6.56	−3.17	p=0.2141

^∗^ p<0.10, ^∗∗^ p<0.05, ^∗∗∗^ *p* < 0.01.

a Questions asked to all participants about visits to people who live inside the WEZ.

b Questions asked only to WEZ Residents about visits to people who live outside of the WEZ.

**Table 4:** Differences in the number of visits over time and by treatment status.

	Control group			Treatment group			Difference in difference	
	(1) W2	(2) W4	(3) Diff. (2)-(1)	(4) W2	(5) W4	(6) Diff. (5)-(4)	(7) D-i-D (6)-(3)	(8) p-value†
Visits to family at their home a	33.79	30.59	−3.20	78.05	65.58	−12.47	−9.27	p=0.287
Visits to family away from their home a	26.07	20.05	−6.02	56.04	34.01	−22.03	−16.01^∗∗^	p=0.014
Visits to family at their home b	26.07	23.74	−2.33	58.14	44.82	−13.32	−11.01^∗^	p=0.100
Visits to Family away from their home b	20.04	16.41	−3.63	44.38	19.71	−24.67	−21.04^∗∗∗^	p<0.001
Visits to Friends at their home a	61.65	54.21	−7.44	94.94	71.57	−23.37	−15.93^∗∗^	p=0.039
Visits to Friends away from their home a	67.83	57.40	−10.43	101.14	69.67	−31.47	−21.04^∗∗∗^	p=0.006
Visits to Friends at their home b	39.08	33.16	−5.92	61.22	48.75	−12.47	−6.55	p=0.333
Visits to Friends away from their home b	42.87	32.88	−9.99	60.34	37.51	−22.83	−12.84^∗^	p=0.073
Visits as Carer a	15.75	11.65	−4.1	154.02	72.24	−81.78	−77.68^∗∗∗^	p<0.001
Visits as Carer b	4.99	5.45	0.46	149.43	47.32	−102.11	−102.57^∗∗∗^	p<0.001

^∗^ p<0.10, ^∗∗^ p<0.05, ^∗∗∗^ *p* < 0.01.

a Questions asked to all participants about visits to people who live inside the WEZ.

b Questions asked only to WEZ Residents about visits to people who live outside of the WEZ.

† p-Values are calculated from regression *Y*_*imt*_ = *α* + *β*(Car_*m*_) + *γ*(Post_*t*_) + *δ*(Car_*m*_ × Post_*t*_) + *ε*_*imt*_ with no additional covariates.

**Table 5:** The effect of the WEZ on the frequency of visits to family: OLS results.

	Visits to family at their home				Visits to family away from their home			
Visit type:	All to R (1)	R to R (2)	N to R (3)	R to N(4)	All to R (5)	R to R (6)	N to R (7)	R to N (8)
After WEZ implementation	−4.330	−13.27^∗∗∗^	4.384	−3.026	−6.341^∗∗∗^	−11.04^∗∗∗^	−1.846	−4.229
	(2.677)	(4.752)	(2.993)	(2.878)	(2.198)	(3.927)	(2.446)	(2.604)

Uses car to visit family	45.06^∗∗∗^	16.40^∗^	86.82^∗∗∗^	32.80^∗∗∗^	30.80^∗∗∗^	12.12^∗^	58.68^∗∗∗^	24.50^∗∗∗^
	(7.345)	(8.771)	(14.809)	(5.412)	(5.558)	(6.661)	(12.152)	(4.877)

After x Car user	−11.69	0.687	−36.63^∗∗∗^	−12.05^∗∗^	−16.81^∗∗^	−12.28	−22.41	−22.48^∗∗∗^
	(7.128)	(8.899)	(13.376)	(5.516)	(6.542)	(7.623)	(15.336)	(5.324)

Employer	−39.59^∗∗∗^	−36.46^∗∗∗^	−32.26^∗∗∗^	−3.912	−18.96^∗∗∗^	−15.54^∗^	−14.18^∗∗^	−3.374
	(8.188)	(12.202)	(9.358)	(10.404)	(5.845)	(9.049)	(5.977)	(8.337)

Employee	−23.62^∗∗∗^	−16.02^∗^	−18.23^∗∗^	−3.720	−3.415	2.406	−0.333	−2.101
	(6.893)	(9.443)	(8.820)	(5.758)	(4.761)	(6.858)	(5.502)	(4.127)

Self-employed	−10.82	−8.805	−4.646	−4.701	2.265	3.652	6.318	−0.0856
	(7.402)	(9.778)	(9.156)	(5.702)	(4.948)	(6.499)	(6.507)	(4.117)

Full-time student	12.29	28.38	−5.843	4.099	12.31	24.15	1.041	−0.926
	(13.459)	(24.077)	(12.044)	(9.106)	(10.184)	(19.613)	(9.041)	(7.868)

Other employment	21.10	24.23	2.172	2.500	4.225	0.415	7.710	6.065
	(16.685)	(19.761)	(30.271)	(10.197)	(10.675)	(13.629)	(14.423)	(7.583)

Income increased since wave 2	−1.184	2.381	−3.676	−0.699	0.737	7.433	−3.923	−0.0255
	(6.387)	(11.308)	(6.898)	(6.157)	(4.505)	(8.528)	(4.647)	(4.341)

Income decreased since wave 2	13.71^∗^	23.93^∗^	4.001	7.852	4.186	5.444	2.810	8.480
	(8.146)	(12.722)	(9.816)	(7.924)	(5.492)	(7.876)	(7.383)	(5.550)

Age 18–24	28.02^∗∗^	77.28^∗∗∗^	10.53	6.334	15.92^∗^	38.90^∗∗^	7.373	21.09^∗∗^
	(12.734)	(26.063)	(12.457)	(11.678)	(8.775)	(17.247)	(8.929)	(10.088)

Age 25–44	4.806	2.921	8.379	−7.329	3.985	4.228	3.547	1.745
	(7.790)	(10.530)	(10.110)	(6.102)	(5.169)	(7.424)	(5.881)	(4.146)

Age 45–59	5.424	7.680	0.793	−2.439	3.521	6.801	−2.896	2.907
	(7.713)	(10.181)	(9.513)	(6.557)	(5.294)	(7.206)	(6.239)	(4.335)

Age 60–64	6.599	18.28	−9.329	4.980	6.590	13.98^∗^	−4.435	10.31
	(9.133)	(12.539)	(10.621)	(8.086)	(5.760)	(7.776)	(6.794)	(6.339)

Male	−8.849^∗∗^	−9.750	−5.551	−2.101	−1.135	0.501	−0.727	0.496
	(4.008)	(6.706)	(3.985)	(3.805)	(2.982)	(4.899)	(3.267)	(2.818)

Constant	44.13^∗∗∗^	56.51^∗∗∗^	26.14^∗∗∗^	30.80^∗∗∗^	23.01^∗∗∗^	29.39^∗∗∗^	14.48^∗∗∗^	16.94^∗∗∗^
	(6.205)	(8.245)	(8.239)	(4.567)	(4.014)	(5.643)	(4.968)	(3.227)

Observations	1982	988	994	1125	1994	990	1004	1122

Standard errors in parentheses. ^∗^ p<0.10, ^∗∗^ p<0.05, ^∗∗∗^ *p* < 0.01.

For visit type: ‘R’ represents WEZ-residents, ‘N’ is non-residents, and ‘All’ is the full sample (residents  + non-residents).

**Table 6:** The effect of the WEZ on the frequency of visits to friends: OLS results.

	Visits to family at their home				Visits to family away from their home			
Visit type:	All to R (1)	R to R (2)	N to R (3)	R to N(4)	All to R (5)	R to R (6)	N to R (7)	R to N (8)
After WEZ implementation	−5.546^∗^	−4.798	−5.409^∗^	−4.869	−9.107^∗∗∗^	−10.97^∗∗^	−7.696^∗∗^	−10.48^∗∗∗^
	(2.899)	(4.993)	(3.230)	(3.080)	(2.908)	(4.848)	(3.586)	(3.391)

Uses car to visit friends	33.70^∗∗∗^	20.42^∗∗∗^	27.31^∗∗∗^	24.16^∗∗∗^	33.84^∗∗∗^	20.36^∗∗∗^	34.77^∗∗∗^	17.91^∗∗∗^
	(5.551)	(7.684)	(6.863)	(5.109)	(5.865)	(7.788)	(8.916)	(5.470)

After x Car user	−17.22^∗∗∗^	−17.04^∗∗^	−17.89^∗∗^	−8.264	−21.63^∗∗∗^	−13.66^∗^	−34.57^∗∗∗^	−13.55^∗∗^
	(6.047)	(8.404)	(8.370)	(5.376)	(6.204)	(8.226)	(9.689)	(6.175)

Employer	−32.40^∗∗∗^	−4.899	−27.20^∗∗∗^	−2.719	−27.36^∗∗^	−9.055	−25.85^∗^	−10.01
	(11.010)	(18.493)	(8.351)	(11.669)	(11.887)	(19.097)	(13.385)	(9.117)

Employee	−38.75^∗∗∗^	−32.30^∗∗∗^	−16.45^∗∗^	−5.832	−36.59^∗∗∗^	−29.95^∗∗∗^	−25.21^∗∗∗^	−8.719
	(5.820)	(8.235)	(7.085)	(6.135)	(5.775)	(7.999)	(7.556)	(5.999)

Self-employed	−14.16^∗∗^	−7.394	−4.126	−2.989	−9.450	1.119	−11.03	3.360
	(6.392)	(8.466)	(7.933)	(5.347)	(6.528)	(8.562)	(8.486)	(5.580)

Full-time student	−12.10	−13.18	−3.670	36.78^∗^	−7.055	5.776	−14.56	23.24
	(10.886)	(17.413)	(12.216)	(19.022)	(10.906)	(16.898)	(12.940)	(16.628)

Other employment	5.528	17.24	−29.43^∗∗∗^	3.115	−20.29^∗^	−12.67	−41.33^∗∗∗^	−5.284
	(13.378)	(15.018)	(8.549)	(10.374)	(10.848)	(13.004)	(10.200)	(8.764)

Income increased since wave 2	−8.933	−13.13	−5.828	−4.759	−3.056	0.469	−2.784	4.763
	(6.166)	(11.599)	(6.354)	(7.855)	(6.277)	(10.441)	(7.355)	(7.619)

Income decreased since wave 2	−3.648	−9.070	5.130	1.133	−5.001	−13.50	6.432	2.923
	(6.693)	(10.715)	(7.254)	(6.599)	(6.086)	(8.949)	(7.926)	(6.994)

Age 18–24	31.06^∗∗∗^	63.37^∗∗∗^	24.89^∗^	11.43	38.18^∗∗∗^	54.86^∗∗∗^	43.72^∗∗∗^	36.72^∗∗^
	(11.910)	(19.888)	(13.786)	(15.587)	(12.203)	(18.561)	(15.067)	(16.534)

Age 25–44	9.236	22.38^∗∗^	6.288	5.580	13.24^∗^	18.09^∗^	20.35^∗∗^	15.97^∗∗∗^
	(6.849)	(9.377)	(8.179)	(6.479)	(7.015)	(9.373)	(8.944)	(6.130)

Age 45–59	4.764	9.759	−0.813	1.812	0.487	1.758	4.828	9.946
	(6.833)	(9.136)	(7.961)	(6.561)	(7.035)	(9.251)	(8.855)	(6.327)

Age 60–64	0.703	7.492	−6.113	0.290	−8.161	−2.615	−10.96	2.944
	(8.063)	(10.539)	(9.389)	(6.484)	(7.715)	(10.163)	(9.025)	(6.033)

Male	−8.057^∗∗^	−6.881	−3.570	5.603	−4.416	4.619	−7.883^∗^	7.982^∗∗^
	(3.770)	(6.028)	(3.930)	(3.883)	(3.810)	(5.873)	(4.320)	(3.963)

Constant	79.23^∗∗∗^	90.58^∗∗∗^	46.76^∗∗∗^	34.01^∗∗∗^	82.17^∗∗∗^	89.72^∗∗∗^	57.17^∗∗∗^	31.08^∗∗∗^
	(5.633)	(7.205)	(7.115)	(4.397)	(5.874)	(7.508)	(8.054)	(3.840)

Observations	2457	1229	1228	1223	2468	1231	1237	1222

Standard errors in parentheses. ^∗^ p<0.10, ^∗∗^ p<0.05, ^∗∗∗^ *p* < 0.01.

For visit type: ‘R’ represents WEZ-residents, ‘N’ is non-residents, and ‘All’ is the full sample (residents  + non-residents).

**Table 7:** The effect of the WEZ on the frequency of visits to act as a carer: OLS results.

Visit type:	All to R (1)	R to R (2)	N to R (3)	R to N (4)
After WEZ implementation	−2.853	−7.257^∗^	1.137	0.595
	(2.292)	(3.723)	(2.751)	(2.088)

Uses car to provide care	140.6^∗∗∗^	131.6^∗∗∗^	155.3^∗∗∗^	146.4^∗∗∗^
	(20.218)	(22.718)	(44.001)	(24.468)

After x Car user	−78.63^∗∗^	−71.28^∗^	−106.4	−104.1^∗∗∗^
(31.918)	(37.997)	(68.661)	(29.937)	

Employer	−18.02^∗∗∗^	−25.24^∗∗∗^	−8.119	4.024
	(5.821)	(9.734)	(5.485)	(9.640)

Employee	−8.555^∗^	−5.128	−3.937	−1.991
	(4.730)	(7.685)	(4.597)	(3.114)

Self-employed	−6.300	−6.198	−2.539	−5.181^∗^
	(4.967)	(6.931)	(5.325)	(3.045)

Full-time student	16.25	31.63	10.49	13.68
	(11.243)	(22.668)	(9.864)	(12.847)

Other employment	14.28	2.412	48.55	1.186
	(12.262)	(12.152)	(36.737)	(5.001)

Income increased since wave 2	−4.981	−11.69^∗∗^	−0.995	−1.430
	(4.331)	(5.784)	(6.292)	(3.441)

Income decreased since wave 2	−1.454	4.429	−7.659^∗∗^	2.358
	(4.622)	(8.404)	(3.253)	(4.965)

Age 18–24	−10.00	−16.71	−1.269	−9.776
	(7.796)	(17.806)	(6.753)	(9.089)

Age 25–44	5.557	5.525	11.13^∗^	0.0885
	(5.276)	(7.500)	(6.043)	(2.885)

Age 45–59	6.868	7.881	10.48^∗^	−1.077
	(5.281)	(7.373)	(5.570)	(2.958)

Age 60–64	−1.900	−2.875	4.525	2.811
	(6.163)	(9.501)	(5.081)	(5.565)

Male	−4.283	−6.414	−1.254	−2.208
	(3.025)	(5.037)	(3.235)	(2.117)

Constant	18.19^∗∗∗^	25.14^∗∗∗^	1.879	6.842^∗∗∗^
	(4.065)	(5.540)	(3.541)	(2.194)

Observations	1842	891	951	869

Standard errors in parentheses. ^∗^ p<0.10, ^∗∗^ p<0.05, ^∗∗∗^ *p* < 0.01.

For visit type: ‘R’ represents WEZ-residents, ‘N’ is non-residents, and ‘All’ is the full sample (residents + non-residents).

**Table 8:** The effect of the WEZ on the frequency of visits to family: Interval Regression results.

	Visits to family at their home				Visits to family away from their home			
Visit type:	All to R (1)	R to R (2)	N to R (3)	R to N(4)	All to R (5)	R to R (6)	N to R (7)	R to N (8)
After WEZ implementation	−3.805	−12.41^∗∗^	3.985	−2.749	−5.139^∗∗^	−9.638^∗∗^	−1.132	−3.998^∗^
	(3.490)	(6.265)	(3.201)	(3.095)	(2.444)	(4.326)	(2.495)	(2.186)

Uses car to visit family	42.61^∗∗∗^	16.17^∗∗^	79.97^∗∗∗^	28.37^∗∗∗^	27.07^∗∗∗^	11.71^∗∗^	49.56^∗∗∗^	19.13^∗∗∗^
	(5.548)	(8.111)	(7.637)	(4.119)	(3.935)	(5.639)	(5.996)	(2.887)

After x Car user	−11.72	−0.000536	−35.74^∗∗∗^	−10.54^∗^	−15.48^∗∗∗^	−11.62	−21.18^∗∗^	−17.25^∗∗∗^
	(7.915)	(11.559)	(10.689)	(5.782)	(5.607)	(8.038)	(8.428)	(4.043)

Employer	−35.62^∗∗∗^	−33.73^∗∗^	−26.75^∗∗∗^	−3.665	−16.06^∗∗^	−14.09	−10.47	−3.003
	(9.124)	(15.931)	(8.935)	(8.561)	(6.521)	(11.081)	(7.152)	(5.961)

Employee	−21.44^∗∗∗^	−14.92^∗∗^	−15.46^∗∗∗^	−3.361	−3.087	1.925	−0.212	−1.452
	(4.545)	(7.393)	(5.106)	(3.722)	(3.194)	(5.132)	(3.968)	(2.618)

Self-employed	−9.903^∗∗^	−8.247	−3.627	−4.030	1.600	2.902	5.172	0.287
	(4.825)	(7.234)	(5.636)	(3.618)	(3.386)	(5.022)	(4.362)	(2.545)

Full-time student	11.81	27.85^∗^	−5.398	3.717	11.94^∗∗^	22.57^∗∗^	2.804	−1.073
	(8.295)	(15.103)	(8.018)	(7.472)	(5.816)	(10.502)	(6.219)	(5.319)

Other employment	19.49^∗^	23.18	3.063	2.316	3.552	0.686	3.996	4.388
	(10.636)	(14.723)	(14.593)	(7.423)	(7.366)	(9.961)	(11.639)	(5.227)

Income increased since wave 2	−1.349	2.124	−3.367	−0.668	0.749	6.483	−2.417	0.552
	(6.912)	(13.595)	(5.977)	(6.299)	(4.874)	(9.394)	(4.712)	(4.440)

Income decreased since wave 2	12.55^∗∗^	22.47^∗∗^	3.644	6.340	3.214	4.525	2.207	5.571
	(6.241)	(10.485)	(6.091)	(5.304)	(4.402)	(7.300)	(4.764)	(3.700)

Age 18–24	26.50^∗∗∗^	76.84^∗∗∗^	9.568	5.574	13.54^∗∗^	36.60^∗∗∗^	5.900	15.93^∗∗^
	(8.654)	(17.458)	(8.298)	(8.937)	(6.088)	(12.321)	(6.426)	(6.405)

Age 25–44	4.600	2.753	7.834	−5.654	3.224	3.247	2.634	0.977
	(5.279)	(8.097)	(6.239)	(4.042)	(3.705)	(5.614)	(4.833)	(2.832)

Age 45–59	5.046	7.227	1.283	−1.955	2.978	5.839	−2.345	1.806
	(5.233)	(7.723)	(6.377)	(3.904)	(3.671)	(5.343)	(4.937)	(2.742)

Age 60–64	5.936	17.29^∗^	−7.139	4.809	5.301	12.23^∗^	−3.687	7.678^∗∗^
	(6.096)	(9.153)	(7.119)	(4.595)	(4.297)	(6.367)	(5.549)	(3.236)

Male	−8.044^∗∗∗^	−9.253^∗^	−4.578	−2.165	−0.812	0.298	−0.345	−0.0896
	(3.054)	(5.270)	(2.918)	(2.600)	(2.147)	(3.653)	(2.278)	(1.830)

Constant	39.00^∗∗∗^	51.87^∗∗∗^	20.54^∗∗∗^	25.48^∗∗∗^	18.78^∗∗∗^	25.13^∗∗∗^	10.86^∗∗^	13.70^∗∗∗^

	(4.481)	(6.735)	(5.507)	(3.307)	(3.129)	(4.631)	(4.269)	(2.329)
Observations	1982	988	994	1125	1994	990	1004	1122

Standard errors in parentheses. ^∗^ p<0.10, ^∗∗^ p<0.05, ^∗∗∗^ *p* < 0.01.

For visit type: ‘R’ represents WEZ-residents, ‘N’ is non-residents, and ‘All’ is the full sample (residents + non-residents).

**Table 9:** The effect of the WEZ on the frequency of visits to friends: Interval Regression results.

	Visits to family at their home				Visits to family away from their home			
Visit type:	All to R (1)	R to R (2)	N to R (3)	R to N(4)	All to R (5)	R to R (6)	N to R (7)	R to N (8)
After WEZ implementation	−5.262^∗^	−4.701	−4.884^∗^	−3.981	−8.573^∗∗∗^	−10.55^∗∗^	−6.898^∗∗^	−9.079^∗∗∗^
	(2.739)	(4.899)	(2.809)	(2.600)	(2.761)	(4.732)	(3.241)	(2.918)

Uses car to visit friends	33.88^∗∗∗^	21.23^∗∗∗^	25.79^∗∗∗^	21.76^∗∗∗^	34.16^∗∗∗^	21.18^∗∗∗^	34.00^∗∗∗^	16.36^∗∗∗^
	(5.449)	(7.642)	(6.303)	(4.576)	(5.783)	(7.742)	(8.555)	(4.911)

After x Car user	−17.67^∗∗∗^	−17.44^∗∗^	−17.30^∗∗^	−8.023^∗^	−22.10^∗∗∗^	−14.31^∗^	−33.01^∗∗∗^	−12.39^∗∗^
	(5.887)	(8.308)	(7.517)	(4.718)	(6.063)	(8.141)	(9.102)	(5.475)

Employer	−31.62^∗∗∗^	−5.166	−23.61^∗∗∗^	−2.406	−27.17^∗∗^	−9.411	−24.79^∗∗^	−8.692
	(10.477)	(18.477)	(7.351)	(10.179)	(11.324)	(18.898)	(12.031)	(7.707)

Employee	−37.28^∗∗∗^	−32.03^∗∗∗^	−14.37^∗∗^	−5.147	−35.34^∗∗∗^	−29.43^∗∗∗^	−23.52^∗∗∗^	−7.575
	(5.649)	(8.117)	(6.349)	(5.289)	(5.593)	(7.843)	(7.082)	(5.196)

Self-employed	−13.99^∗∗^	−7.388	−3.631	−2.446	−9.370	1.335	−10.64	3.293
	(6.203)	(8.368)	(7.067)	(4.597)	(6.325)	(8.423)	(7.885)	(4.844)

Full-time student	−11.25	−12.35	−2.739	34.60^∗^	−6.200	7.050	−13.24	22.40
	(10.557)	(17.343)	(11.005)	(17.979)	(10.711)	(17.111)	(12.136)	(15.420)

Other employment	5.557	18.19	−24.40^∗∗∗^	1.903	−19.53^∗^	−11.84	−36.82^∗∗∗^	−4.611
	(13.096)	(15.026)	(7.344)	(8.763)	(10.475)	(12.877)	(9.184)	(7.445)

Income increased since wave 2	−8.442	−13.00	−4.955	−3.662	−3.071	0.646	−2.568	4.435
	(5.714)	(11.106)	(5.398)	(6.626)	(5.901)	(10.224)	(6.487)	(6.597)

Income decreased since wave 2	−3.686	−9.467	4.592	0.422	−4.824	−13.29	5.657	1.804
	(6.361)	(10.411)	(6.366)	(5.556)	(5.805)	(8.717)	(7.167)	(5.987)

Age 18–24	29.96^∗∗∗^	63.25^∗∗∗^	22.71^∗^	10.11	37.20^∗∗∗^	55.42^∗∗∗^	41.21^∗∗∗^	32.95^∗∗^
	(11.528)	(20.027)	(12.592)	(14.079)	(11.941)	(18.986)	(14.103)	(15.661)

Age 25–44	9.017	22.34^∗∗^	5.801	4.662	13.03^∗^	18.14^∗∗^	19.49^∗∗^	13.71^∗∗^
	(6.631)	(9.246)	(7.213)	(5.583)	(6.809)	(9.239)	(8.249)	(5.334)

Age 45–59	4.647	9.790	−0.643	1.526	0.754	1.873	5.163	8.819
	(6.609)	(8.993)	(6.969)	(5.679)	(6.791)	(9.034)	(8.106)	(5.489)

Age 60–64	0.376	7.146	−4.548	0.185	−7.714	−2.526	−8.784	3.043
	(7.721)	(10.359)	(8.071)	(5.473)	(7.379)	(9.910)	(8.120)	(5.169)

Male	−7.735^∗∗^	−6.932	−2.995	4.722	−4.426	4.398	−7.161^∗^	6.755^∗^
	(3.579)	(5.908)	(3.409)	(3.370)	(3.635)	(5.762)	(3.908)	(3.476)

Constant	73.81^∗∗∗^	86.27^∗∗∗^	39.72^∗∗∗^	28.66^∗∗∗^	76.67^∗∗∗^	84.93^∗∗∗^	50.59^∗∗∗^	26.26^∗∗∗^
	(5.499)	(7.114)	(6.324)	(3.910)	(5.737)	(7.406)	(7.426)	(3.361)

Observations	2457	1229	1228	1223	2468	1231	1237	1222

Standard errors in parentheses. ^∗^ p<0.10, ^∗∗^ p<0.05, ^∗∗∗^ *p* < 0.01.

For visit type: ‘R’ represents WEZ-residents, ‘N’ is non-residents, and ‘All’ is the full sample (residents + non-residents).

**Table 10:** The effect of the WEZ on the frequency of visits to act as a carer: Interval Regression results.

Visit type:	All to R (1)	R to R (2)	N to R (3)	R to N (4)
After WEZ implementation	−2.359	−6.279	1.030	0.504
	(2.381)	(4.163)	(2.485)	(1.809)

Uses car to provide care	139.3^∗∗∗^	130.5^∗∗∗^	157.4^∗∗∗^	150.8^∗∗∗^
	(10.273)	(14.062)	(15.940)	(7.465)

After x Car user	−84.44^∗∗∗^	−75.83^∗∗∗^	−114.9^∗∗∗^	−118.3^∗∗∗^
	(15.041)	(21.067)	(22.589)	(10.653)

Employer	−15.32^∗∗^	−21.82^∗^	−7.038	2.941
	(7.002)	(13.063)	(7.306)	(5.641)

Employee	−7.127^∗∗^	−4.477	−3.192	−1.765
	(3.383)	(5.592)	(4.190)	(2.427)

Self-employed	−5.332	−5.431	−2.219	−3.980^∗^
	(3.571)	(5.470)	(4.534)	(2.397)

Full-time student	13.71^∗∗^	28.17^∗∗^	6.774	12.56^∗∗∗^
	(6.059)	(11.141)	(6.432)	(4.745)

Other employment	11.93	1.688	41.95^∗∗∗^	0.966
	(7.565)	(10.536)	(11.682)	(4.574)

Income increased since wave 2	−4.537	−10.35	−1.236	−1.223
	(5.015)	(9.447)	(4.974)	(4.373)

Income decreased since wave 2	−1.344	3.787	−6.407	1.251
	(4.628)	(7.999)	(4.896)	(3.456)

Age 18–24	−8.079	−14.75	0.148	−8.483
	(6.428)	(12.675)	(6.913)	(5.514)

Age 25–44	5.104	5.096	9.835^∗^	0.468
	(3.915)	(6.084)	(5.158)	(2.648)

Age 45–59	5.960	6.963	8.808^∗^	−0.274
	(3.845)	(5.733)	(5.212)	(2.497)

Age 60–64	−1.688	−2.683	3.815	3.155
	(4.405)	(6.766)	(5.685)	(2.959)

Male	−3.474	−5.523	−0.790	−1.060
	(2.236)	(3.941)	(2.335)	(1.717)

Constant	14.79^∗∗∗^	21.43^∗∗∗^	0.800	4.405^∗∗^
	(3.107)	(4.564)	(4.445)	(1.972)

Observations	1842	891	951	869

Standard errors in parentheses. ^∗^ p<0.10, ^∗∗^ p<0.05, ^∗∗∗^ *p* < 0.01.

For visit type: ‘R’ represents WEZ-residents, ‘N’ is non-residents, and ‘All’ is the full sample (residents + non-residents).

**Table 11:** Probability of using each mode in wave 4, given journeys were made by car in wave 2.

Outcome	Car	Pub. trans.	Taxi	Walk	Cycle
Visits to family at their home a	52.08%	31.25%	0.00%	16.67%	0.00%
Visits to family away from their home a	44.44%	33.33%	7.41%	14.81%	0.00%
Visits to family at their home b	75.26%	23.71%	0.82%	0.82%	0.00%
Visits to family away from their home b	60.42%	31.25%	2.08%	4.17%	2.08%
Visits to friends at their home a	57.43%	25.74%	0.00%	16.83%	0.00%
Visits to friends away from their home a	47.47%	34.34%	3.03%	14.14%	1.01%
Visits to friends at their home b	68.00%	30.40%	0.00%	1.60%	0.00%
Visits to friends away from their home b	59.74%	31.17%	2.60%	5.19%	1.30%
Visits as carer a	75.29%	0.00%	0.00%	24.71%	0.00%
Visits as carer b	100%	0.00%	0.00%	0.00%	0.00%

a questions asked to all participants about visits to people who live inside the WEZ.

b questions asked only to WEZ Residents about visits to people who live outside of the WEZ.

## Footnotes

1 Using questions such as: *“ Do you think someone living nearby would help you in an emergency? ”*.
